# Epitope-Specific Anti-SerpinB3 Antibodies for SerpinB3 Recognition and Biological Activity Inhibition

**DOI:** 10.3390/biom13050739

**Published:** 2023-04-25

**Authors:** Alessandra Biasiolo, Michele Sandre, Stefania Ferro, Santina Quarta, Mariagrazia Ruvoletto, Gianmarco Villano, Cristian Turato, Maria Guido, Oriano Marin, Patrizia Pontisso

**Affiliations:** 1 Department of Medicine, University of Padova, Via Giustiniani 2, 35128 Padova, Italy; alessandra.biasiolo@unipd.it (A.B.); santina.quarta@unipd.it (S.Q.); mariagrazia.ruvoletto@unipd.it (M.R.); mguido@unipd.it (M.G.); 2 Department of Biomedical Sciences, University of Padova, Via Colombo 3, 35131 Padova, Italy; michele.sandre@unipd.it (M.S.); stefania.ferro@unipd.it (S.F.); oriano.marin@unipd.it (O.M.); 3 Department of Surgical, Oncological and Gastroenterological Sciences, University of Padova, Via Giustiniani 2, 35128 Padova, Italy; gianmarco.villano@unipd.it; 4 Department of Molecular Medicine, University of Pavia, 27100 Pavia, Italy; cristian.turato@unipv.it

**Keywords:** SerpinB3, SerpinB4, immunogenic epitopes, serine protease inhibitory activity

## Abstract

SerpinB3 is a serine protease inhibitor that plays a relevant role in disease progression and cancer by increasing fibrosis, cell proliferation, and invasion, besides conferring resistance to apoptosis. The mechanisms underlying these biological activities are not yet fully understood. The aim of this study was to generate antibodies directed against different SerpinB3 epitopes to better investigate their biological role. Five exposed epitopes were identified using the software DNASTAR Lasergene and the corresponding synthetic peptides were used for NZW rabbit immunization. Anti-P#2 and anti-P#4 antibodies were able to recognize both SerpinB3 and SerpinB4 by ELISA. Anti-P#5 antibody, produced against the reactive site loop of SerpinB3, showed the greatest specific reactivity for human SerpinB3. This antibody was able to recognize SerpinB3 at nuclear level, while anti-P#3 antibody recognized SerpinB3 only at cytoplasmic level, both by immunofluorescence and by immunohistochemistry. The biological activity of each antibody preparation was assessed in HepG2 cells overexpressing SerpinB3 and anti-P#5 antibody reduced proliferation by 12% cell and cell invasion by 75%, while trivial results were obtained with the other antibody preparations. These findings indicate that the reactive site loop of SerpinB3 is essential for the invasiveness features induced by this serpin and it could become a novel druggable target.

## 1. Introduction

The protease inhibitor SerpinB3, previously known as squamous cell carcinoma antigen (SCCA1), belongs to the serine protease inhibitor (Serpin) superfamily. These proteins share a ternary structure consisting of an ovalbumin-like domain with nine α-helices, three β-sheets, and a reactive site loop (RSL, also known as a reactive center loop; RCL) [1,2,3].

In humans, the two isoforms SerpinB3 (SB3) and SerpinB4 (SB4), encoded by genes located on the long arm of chromosome 18 (18q21.3), consist of 390 amino acids with 92% homology at the amino acid level. The catalytic site (RLS) shows a minor degree of homology, with only 7 amino acids out of 13 identical (54%). This accounts for the different targets of SB3 and SB4. The first is known to inhibit papain-like cysteine proteases, while SB4 mainly inhibits chymotrypsin-like serine proteases. In mice, the locus (on chromosome 1D) is amplified to include four genes (*Serpinb3a*, *b3b*, *b3c*, and *b3d*) and three pseudogenes. Serpinb3a most closely resembles SB4 and SB3 [4] and targets both serine and cysteine-like proteases [5], serving the function of SB3/4 isoforms.

Originally discovered in 1970s by Kato and Torigoe in squamous cell carcinoma of the cervix, the SerpinB3/4 isoforms have been detected in the immune, nervous, muscular, secretory, and reproductive systems and in internal organs [6,7,8]. Upregulation of SB3/4 has been reported in several types of cancer [9,10,11,12,13,14,15,16,17,18]. Although it was initially reported that the two isoforms are cytosolic proteins, passively released from dying cells [19], additional cytoplasmic and nuclear localizations have been subsequently described [20]. SB3/4 isoforms circulate in serum, free or linked with natural IgM to form immune complexes [21] and are associated with disease progression and cancer development. In different tumors and skin diseases, different expression of the two isoforms has been detected and their combined measurement was described as a useful tool for differential diagnosis [13] and prognosis [22]. In particular, SB3 was identified as a relevant player in carcinogenesis, and high expression of this molecule was associated with poor prognosis in liver, colon and esophageal cancer [11,23,24,25,26]. Due to the pivotal role of this serpin, the demand for routine clinical testing has rapidly increased. Common methods used to detect the protein expression of this serpin in serum and in tissue samples include enzyme-linked immunosorbent assay (ELISA), Western blot (WB), immunochemistry (IHC) and immunoluminometric assay. To date, the main limitation for the use of SerpinB3 detection in clinical practice regards the poor performance of available assays, lacking sensitivity and specificity, mainly due to shared antibody reactivity for both SB3/4 isoforms. This fact could explain, at least in part, the conflicting results obtained regarding the clinical value of SB3/4 as a biomarker [27], besides other methodological heterogeneity, including the type of assay and the considered cutoff. The purpose of the present study was to develop antibodies with high affinity and specificity towards SB3 immunogenic epitopes using SB3 synthetic peptides as immunogens for the development of immuno-based analytical methods and to better identify target SB3 regions for the development of immunotherapeutic strategies.

## 2. Methods

### 2.1. Peptide Synthesis

Peptides were designed using the software DNASTAR Lasergene. After the submission of SerpinB3 primary protein sequence (UNIPROT: P29508) to the software, the antigenicity plot was analyzed and compared with tridimensional structure (PDB: 2ZV6) in order to identify the most exposed epitopes. Finally, the sorted epitopes were composed of 5 peptides along all the protein structure: peptide #1 (aa 16–30), peptide #2 (aa 140–154), peptide #3 (aa 268–279), peptide #4 (aa 191–209), and peptide #5 (aa 340–368). Peptide #5 encompasses all amino acids of the loop responsible for the antiprotease activity of SerpinB3.

To promote KLH conjugation (see below), a dipeptide Cys-β-Ala was added to the N terminus tail of peptides #1, #2, #3 and #4 (See Supplementary Materials Figure S1B). They were prepared by solid-phase peptide synthesis using a multiple peptide synthesizer (Syro II, Biotage) on *p*-benzyloxybenzyl alcohol resins (Wang resin) or 2-chlorotrityl chloride resin for the synthesis of peptide #5, loaded with their C-terminal amino acids (Novabiochem, Bad Soden, Germany). The fluoren-9-ylmethoxycarbonyl (Fmoc) strategy [28] was used throughout the peptide chain assembly, utilizing *O*-(7-azabenzotriazol-1-yl)-*N*,*N*,*N*′,*N*′-tetramethyluronium hexafluorophosphate (HATU) as coupling reagent [29]. The side-chain-protected amino acid building blocks used were: *N*-α-Fmoc-Nω-(2,2,4,6,7-pentamethyldihydrobenzofuran-5-sulfonyl)-l-arginine, *N*-α-Fmoc-γ-*tert*-butyl-l-glutamic acid, *N*-α-Fmoc-β-*tert*-butyl-l-aspartic acid, *N*-α-Fmoc-*O*-*tert*-butyl-l-tyrosine, *N*-α-Fmoc-*O*-*tert*-butyl-l-serine, *N*-α-Fmoc-*N*ε-(*tert*-butyloxycarbonyl)-l-lysine, *N*-α-Fmoc-*N*(*im*)-trityl-l-histidine, *N*-α-Fmoc-*N*-γ-trityl-l-glutamine, *N*-α-Fmoc-*S*-trityl-cystine,*N*--Fmoc-2,5,7-tri-tert-butyl-L-tryptophan and *N*-α-Fmoc-*N*-β-trityl-l-asparagine,*N*-α-Fmoc-*O*-*tert*-butyl-l-threonine. Cleavage of the peptide was performed by reacting the peptidyl-resins with following mixture 82.5% TFA, 5% phenol, 5% H_2_O, 5% thioanisole and 2.5% EDT (Reagent K) for 2.5 h. Crude peptide was purified by a preparative reverse phase HPLC. Molecular masses of the peptides were confirmed by mass spectroscopy on a MALDI TOF–TOF mass spectrometer [model 4800, Applied Biosystems (Carlsbad, CA, USA). Peptide purity range was 90–95%, as evaluated by analytical reverse-phase HPLC.

### 2.2. Oligoclonal Region-Specific SB3 Antibody Production and Purification

Taking advantage of the sulfhydryl moiety, each synthetic peptide reproducing the immunogenic SB3 regions was conjugated with maleimide activated KLH protein carrier (Sigma Aldrich, St. Louis, MO, USA). The polyclonal antibodies were injected into 3 NZW female rabbits (Envigo, Indianapolis, IN, USA), immunizing two rabbits against a pair of peptides (peptide #1 + #2 and peptide #3 + #4 respectively) and one rabbit against peptide #5. The reason for not performing separate immunizations was dictated mainly by the need to reduce the number of animals used in experiments in accordance with national and European ethics guidelines. The animal study was approved by the Italian Ministry of Health (authorization 99/2020-PR).

Rabbits were injected subcutaneously four times at intervals of 21 days, with 0.5 mg of protein conjugates in PBS, emulsified with Freund’s adjuvant (1:1 *v*/*v*). Starting from the second immunization, 10 mL of blood was collected from the ear vein of each rabbit 15 days after the immune boost. Serum was isolated by centrifugation (3500 rpm, 10 min) and antiserum was purified using an immobilized peptide affinity resin (Sulfo Link Coupling Gel, Thermo Scientific, Waltham, MA, USA) according to manufacturer’s instructions. Briefly, each peptide was coupled, through its Cys residues, to the resin and loaded into a polypropylene column arranging a total of 5 columns. Fractions were eluted with glycine buffer (0.2 M, pH 2.5) into Tris buffer (1 M, pH 8.5) solution for pH neutralization. Protein-eluted fractions (as determined by Abs at 280 nm) were pooled, concentrated by ultrafiltration (Amicon Ultra-15 30KD, Millipore, Burlington, MA, USA) and stored at −80 °C until further analysis.

### 2.3. Biotinylation of Region-Specific SB3 Antibodies

Biotin-labeled antibodies were obtained using a biotinylation kit (Abcam, Cambridge, UK) and following the manufacturer’s instructions. Briefly, 10 μL of modifier reagent solution was added to 50 μL of antibody solution (2 mg/mL in PBS) and gently mixed. The resulting solution was added directly to the Biotin Mix powder and incubated for 2 h in the dark at room temperature (20–25 °C). In the end, 10 μL of Quencher Reagent solution was added to stop the reaction. The biotinylated antibody solution was used without any further purification.

### 2.4. Dot-Blot

Validation of antibody specificity and absence of cross-reactivity between rabbits’ sera were assessed by dot blot against all peptides. Peptides were spotted in quadruplicate on nitrocellulose membrane (0.22 µm pore size, Millipore, Burlington, MA, USA) from 2 to 0.002 µg and then dried. The membranes were incubated with 5% BSA in TBS–Tween 0.05% (TBS-T) for 1 h at room temperature in agitation to block nonspecific sites. After three TBS-T washes, membranes were incubated overnight with affinity-purified rabbit anti-SB3 peptide antibodies (1:1000 in BSA 5%), washed 3 times and then probed for 45 min with HRP-conjugated secondary antibody (Sigma Aldrich, St. Louis, MO, USA) at 1:10,000 dilution. After washes with TBS-T, membranes were developed using enhanced chemiluminescent substrate (ECL advance, Amersham Biosciences, Chicago, IL, USA) and the intensity of signal area was detected by Imager CHEMI Premium (VWR). As control, human recombinant SB3 wild-type (r-SerpinB3) and the SB3 deleted of 7 aa in the catalytic site (∆7-SB3) isoform (0.4–0.04–0.004 µg) were also spotted onto the membranes.

### 2.5. Direct ELISA

The specific reactivity of the antibodies against SB3 peptides was assessed against the human SerpinB3/4 isoforms, the Δ7-SB3 and against murine Serpinb3a using an enzyme-linked immunosorbent assay (ELISA). Human r-SerpinB3 and Δ7-SB3 were obtained in our laboratory, as previously described [30], SB4 was purchased from OriGene Technologies (Rockville, MD, USA) and murine Serpinb3a was purchased from Nzytech (Lisboa, Portugal). Briefly, 96-well ELISA polystyrene high binding plates (Sigma Aldrich, St. Louis, MO, USA) were coated in duplicate overnight at 4 °C with 100 µL of the different isoforms at 10 µg/mL in carbonate buffer pH 9.6. The plates were then blocked using 5% skimmed milk in PBS and incubated for 2 h at room temperature. The plates were washed with PBS 0.5% Tween 20 and subsequently incubated for 1 h at room temperature with the rabbit anti-SB3 peptide antibodies (100 µL) at 5 µg/mL concentration, previously found to be the optimal concentration for SB3 detection with the different antibody preparations. Washing steps were repeated (6×) and the plates were incubated with 100 µL of peroxidase AffiniPure goat anti-rabbit IgG (1:5000; *v*:*v*) (Sigma Aldrich, St. Louis, MO, USA) in similar condition. The plates were then washed (6×) and ELISA reaction was initiated by addition of TMB substrate (100 µL). The reaction was quenched by the addition of 1N HCl (100 µL) and the resulting absorbance was measured with a Victor X3 Microplate reader (Perkin Elmer, Waltham, MA, USA) at 450 nm.

### 2.6. Indirect ELISA

Since anti-SB3peptide #5 was identified as specifically reacting against human SerpinB3, an indirect ELISA was assessed to define the affinity of binding of this antibody, compared to that of a commercially available oligoclonal anti-SB3/4 antibody (Xeptagen, Venice, Italy). The rabbit oligoclonal anti-SB3/4 antibody was coated onto microtiter plate at 10 μg/mL concentration in PBS, pH 7.4 by overnight incubation at 4 °C. After washing, 100 μL of different concentrations of human r-SerpinB3 (16 ng/mL–0.5 ng/mL) was incubated for 1 h at room temperature in duplicate. After an hour’s incubation, 100 μL of biotinylated antibody anti-SB3peptide #5 were added and incubated for 1 h at room temperature. Anti-SB3peptide #5 antibody was tested at three different concentrations: 0.5–1.0–1.5 μg/mL. To develop the assay, 100 μL of HRP-conjugated streptavidin solution at 1:3000 dilution was incubated for 1 h at room temperature, followed by the addition of a ready-to-use TMB substrate (Sigma, St. Louis, MO, USA). The colorimetric reaction was stopped with HCl 1 N (100 μL), and optical density (OD) at 450 nm was determined using a Victor X3 microplate reader (Perkin Elmer, Waltham, MA, USA).

### 2.7. SDS-PAGE and Western Blot Analysis

Lanes of 12% polyacrylamide gel were loaded with human r-SerpinB3 (4 ng) under reducing conditions and transferred (1 h 30 min at 390 mA) to a nitrocellulose membrane using a transfer apparatus according to the manufacturer’s protocol (Bio-Rad Laboratories Inc, Hercules, CA, USA). After 60 min incubation with 5% ECL blocking agent (GE Healthcare, Chicago, IL, USA) in 0.1% Tween 20 in 1X PBS (PBST), (Sigma-Aldrich, St. Louis, MO, USA), the membrane was washed once with PBST and cut into six lanes, and each lane was incubated overnight at 4 °C with a different affinity-purified anti-SB3 peptide antibody preparation at 5 μg/mL concentration or with the anti-SB3/4 monoclonal antibody (OriGene Tech., Rockville, MD, USA). Membranes were washed three times for 10 min and incubated with horseradish peroxidase-conjugated anti-rabbit (Sigma-Aldrich, St. Louis, MO, USA, 1:10,000 dilution) or anti-mouse (KPL, SeraCare Company, Gaithersburg, MD, USA, 1:5000 dilution) antibodies for 2 h at room temperature. Antigenic detection was carried out by an enhanced chemiluminescent substrate (Euroclone SpA, Milano, Italy) according to the manufacturer’s protocol and the densitometric analysis was assessed using the VersaDoc Imaging system (Bio-Rad Laboratories, Hercules, CA, USA).

### 2.8. Immunohistochemistry

Immunohistochemistry (IHC) analysis was carried out using the Dako Omnis autostainer (Dako, Glostrup, Denmark), on formalin-fixed, paraffin-embedded samples of 5 human hepatocellular carcinoma (HCC). Squamous cervical mucosa was used as a positive control, while for negative control, the primary antibody was omitted. Serial dilutions (1:50, 1:100, 1:200, 1:400, 1:500, 1:800, and 1:1000) of primary antibodies were obtained to find the optimal concentration in which binding with the antigen was optimized and nonspecific stains minimized. The following concentrations were finally used: anti-SB3 peptide #1 and anti- SB3 peptide #2 = 1:300, anti SB3 peptide #3 and anti-SB3 peptide #4 = 1:200, anti-SB3 peptide #5 = 1:800. Antigen retrieval was performed at pH 6.0. The reference anti-SerpinB3/4 polyclonal antibody (PA5-30164; Invitrogen, Waltham, MA, USA) was used at 1:100 dilution as further control, and in this case, antigen retrieval was carried out at pH 9.

### 2.9. Immunofluorescence

Immunofluorescence studies were carried out on hepatoma cells (HepG2 cell line) (ATCC, Manassas, VA, USA) engineered to overexpress SerpinB3 (HepG2/SB3) by transfection with a plasmid expression vector containing the human SerpinB3 gene (pCDNA3/SB3), and on HepG2/Control cells containing the plasmid vector alone (pcDNA3.1D/V5-His-TOPOTM), obtained as previously described [31].

HepG2 cells were seeded on slides (4 × 10^5^ cells/slide) and cultured for 48 h. Cells were then fixed with 4% paraformaldehyde, permeabilized with 0.4% Tryton X-100 and blocked with 5% goat serum (Invitrogen, Waltham, MA, USA) in PBS containing 1% BSA. Slides were incubated with 2 μg/mL of anti-P#3antibody, anti-P#5antibody and with anti-SerpinB3/4 monoclonal antibody (OriGene Tech., Rockville, MD, USA) at 1:150 dilution for 1 h at room temperature, followed by incubation with the anti-rabbit Alexa-Goat 546 and anti-mouse Alexa-Goat 488 secondary antibodies (Invitrogen Life Technologies, Waltham, MA, USA). Cellular nuclei were counterstained with Dapi (Merck KGaA, Darmstadt, Germany). Slides were mounted with ELVANOL (Merck KGaA, Darmstadt, Germany) and observed under a fluorescence microscope (Axiovert 200M-Apotome.2, Carl Zeiss MicroImaging GmbH, Göttingen, Germany).

### 2.10. Proliferation and Invasion Assays

Real-time cell proliferation was performed in HepG2 cells (HepG2/CTR) and in HepG2 cells manipulated to overexpress SerpinB3 (HepG2/TG) [31] by the xCELLigence DP instrument (ACEA, San Diego, CA, USA) as previously described [32]. Briefly, 40,000 cells/well were seeded on E-plates 16 (Agilent, San Diego, CA, USA) in the presence of medium alone or of the different antibody preparations at 5 μg/mL concentration. Twenty-four hours after seeding, the proliferation index was monitored every 15 min for up to 72 h in 5% CO_2_ atmosphere, using the RTC software (version 2.0, ACEA BioSciences, San Diego, CA, USA). In parallel, an invasion test was performed using the system’s CIM-plate 16 of the xCELLigence system. Matrigel was used as a solid matrix. The cell index of each well was measured for each experiment every 15 min for up to 23 h.

### 2.11. Statistical Analysis

The results are presented as means and standard deviations or as medians. The experiments were repeated at least three times for all the different methodologies. Statistical analysis was performed using the two-tailed *t*-test, and *p* values ≤ 0.05 were considered significant. Data in bar graphs are presented as means ± SD. Data were analyzed by GraphPad Prism 9 (GraphPad Software Inc., San Diego, CA, USA).

## 3. Results

### 3.1. Antibody Generation

As a first step, five experimental epitopes along all the protein SB3 structure and representing the most exposed epitopes were designed using the software DNASTAR Lasergene. Peptide #1 (aa 16–30), peptide #2 (aa 140–154), peptide #3 (aa 268–279), peptide #4 (aa 191–209), peptide #5 (aa 340–368) were synthesized by a solid-phase peptide method, purified by a preparative reverse-phase HPLC and tested by mass spectroscopy to confirm the molecular mass, as reported in the Section 2. Each peptide, except for peptide #5, contains from 11 to 20 amino acids that were considered to be optimal immunogens [33,34].

### 3.2. Antibody Characterization

**Figure 1:**
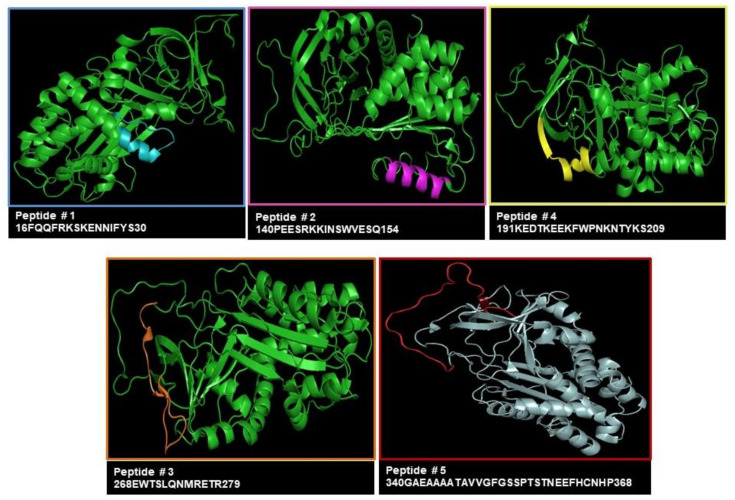
Steric position of SerpinB3 epitopes. Steric position of SerpinB3-exposed epitopes (colored regions) chosen for antibody generation. Each peptide was prepared by solid-phase peptide synthesis method using a multiple peptide synthesizer (Syro II, Biotage, Uppsala, Sweden) on *p*-benzyloxybenzyl alcohol resin (Wang resin).

Figure 1 shows the location of synthetic peptides within the 3D structure of SB3 and the corresponding amino acid sequence. A comparison between the sequence of the different peptides in terms of percentage of identity and number of amino acids overlapping with murine Sb3a sequence was analyzed and is reported in Table 1 and in Supplementary Materials Figure S1. To confirm the specificity, each of three antisera obtained from immunization of animals with a pair of peptides was tested in immuno-dot blot (Supplementary Materials Figure S2). Very low positivity was detectable for antiserum anti-peptide #5 against Δ7-SB3 (SB3 deleted isoform) spotted onto the membrane at 0.4 μg, probably because it was produced against the peptide #5 composed of 30 amino acids, which is bigger than the deleted region of the reactive site of Δ7-SB3 (panel C).

#### 3.2.1. Direct ELISA

**Figure 2:**
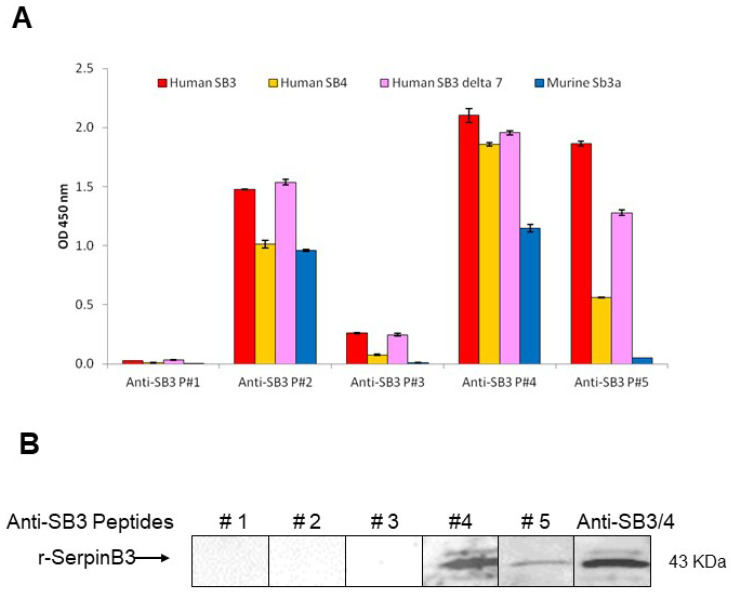
ELISA and Western blot results. (**A**) Direct ELISA results of comparison between binding of antibodies raised against SB3 peptides (#1–#5) with human SB3 wild type, human SB4, human SB3 deleted of the catalytic site (Δ7-SB3) and murine Serpin (Sb3a). The results are expressed as mean ± SD of optical density values (OD) at 450 nm (panel A). (**B**) Western blot analysis of anti-SB3 peptide antibodies reactivity for human SB3 in denaturing conditions. Each lane was loaded with 4 ng/mL of recombinant protein. As control, an anti-SB3/4 monoclonal antibody was used. A reactive band at 43 kDa was detectable using anti-SB3 peptide #4 and anti-SB3 peptide #5 antibodies, similar to that obtained with the reference anti SB3/SB4 antibody. R-SerpinB3, recombinant SerpinB3.

When the antibodies were used in order to recognize the different isoforms of human SB3 and SB4, Δ7-SB3 and murine Sb3a by ELISA, the antibodies anti-peptide #2, #4 and #5 showed moderate/high positivity. Anti-peptide #2 and #4 antibodies recognized well all forms of the serpin, including murine Sb3a, as expected, given the high percentage of identity (92.9% for peptide #2) or the high number of overlapping amino acids in (17aa for peptide #4). Among the five preparations, anti-peptide #4 and anti-peptide #5 antibodies displayed the best reactivity for human SB3, the latter antibody showing trivial reactivity for SB4, at variance with anti-peptide #4; therefore, anti-peptide #5 antibody could be considered SB3-specific (Figure 2A).

#### 3.2.2. Western Blot

Western blot analysis results revealed that in denaturing conditions, only anti-peptide #4 and anti-peptide #5 antibodies were able to recognize r-SerpinB3 immobilized onto nitrocellulose membrane, while the remaining antibodies were not reacting in this experimental setting (Figure 2B).

#### 3.2.3. Indirect ELISA

**Figure 3:**
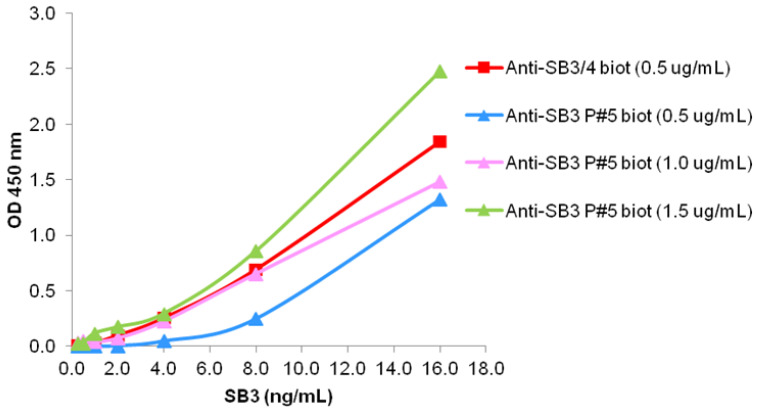
Indirect ELISA. Example of indirect ELISA immunoreactivity of anti-SB3 peptide #5 biotinylated antibody (anti-SB3#5 biot) tested at 0.5–1.0–1.5 μg/mL concentration and of the reference oligoclonal anti SB3/4 biotinylated antibody (anti-SB3/4 biot) tested at 0.5 μg/mL (red square). Serial dilutions of SB3 wild type (range 16 ng/mL–0.25 ng/mL) were coated to the solid phase and the results, expressed as OD values at 450 nm, are presented as median values obtained in three independent experiments.

Since the anti-peptide #5 antibody showed the highest specific reactivity for SB3, we assumed that it could be utilized as secondary antibody in the development of an immuno-based assay for the quantification of free SB3 in serum. This antibody was then conjugated with biotin and the performance of biotinylated anti-peptide #5 antibody was tested towards different concentrations of human recombinant SB3. As shown in Figure 3, this antibody preparation revealed a satisfactory sensitivity when compared to anti SB3/SB4 biotinylated antibody and used at 1 or 1.5 μg/mL concentration (Figure 3).

#### 3.2.4. Imaging Results 

**Figure 4:**
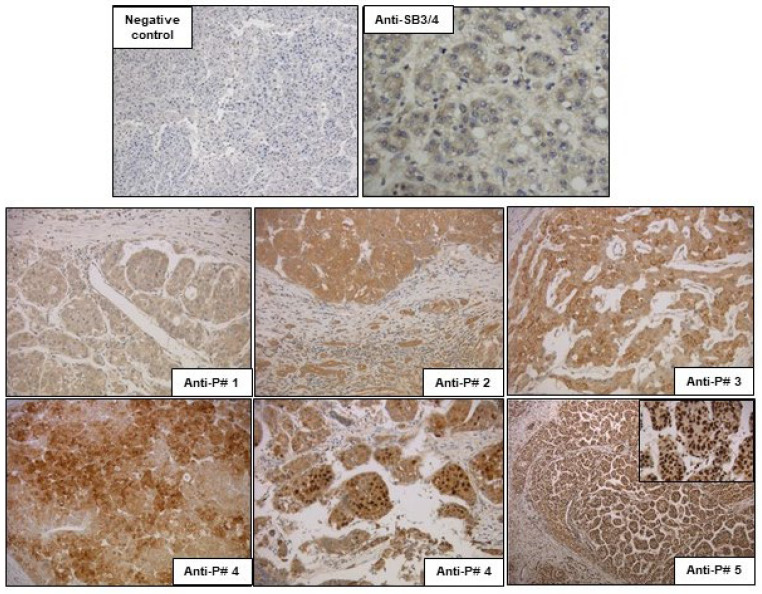
Immunohistochemistry. Examples of anti-SB3 peptide antibodies reactivity by IHC using the reference anti-SB3/4 antibody or the different anti-SB3 peptide (#1–#5) antibody preparations in a paraffin-embedded human specimen of hepatocellular carcinoma. The negative control was generated by the omission of the primary antibody. Magnification of the panels 25×, insert 40×.

**Figure 5:**
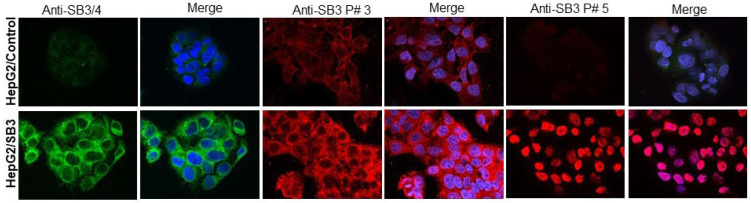
Immunofluorescence results. Example of indirect immunofluorescence analysis for SerpinB3 performed in HepG2 cells overexpressing SerpinB3 (HepG2/SB3) and in HepG2/control cells using anti-SerpinB3/4 commercial antibody as control, anti-SB3 peptide #3 antibody and anti-SB3 peptide #5 antibody. Nuclei are stained in blue (DAPI). Magnification of the panels 63×. Evaluation of immunofluorescence was assessed in three independent experiments examining at least ten random high-power fields.

The anti-peptide antibody preparations were tested to assess their capability to reveal the presence of SB3 in human samples of hepatocellular carcinoma, where this serpin was previously described [16]. As shown in Figure 4, by IHC, very mild expression of SB3 was seen in the cytoplasm of human HCC specimens using anti-P#1 antibody. Anti-P#2 antibody detected cytoplasmic SB3, with moderate intensity. A heterogeneous stain was obtained with anti-P#3 antibody (Figure 4) and with anti-P#4 antibody. With this latter antibody, SB3 reactivity was also detected in some nuclei. Interestingly, a strong diffuse nuclear expression of SB3, in the absence of cytoplasmic stain, was observed using anti-P#5 antibody. The different subcellular distribution of SB3 reactivity detected with the distinct antibody preparations was also confirmed by immunofluorescence in HepG2 cells overexpressing SerpinB3 or in HepG2 control cells, expressing trivial levels of this serpin. Anti-P#3 antibody, showing the best cytoplasmic reactivity by IHC, showed remarkable cytoplasmic reactivity for SB3 in HepG2/SB3 cells, similar to that observed with the reference anti-SB3/4 antibody, while anti-P#5 antibody, showing the best nuclear reactivity by IHC, confirmed also in this case the nuclear localization of the serpin only in cells genetically manipulated to overexpress SerpinB3 (Figure 5).

### 3.3. Antibody Biological Activity

**Figure 6:**
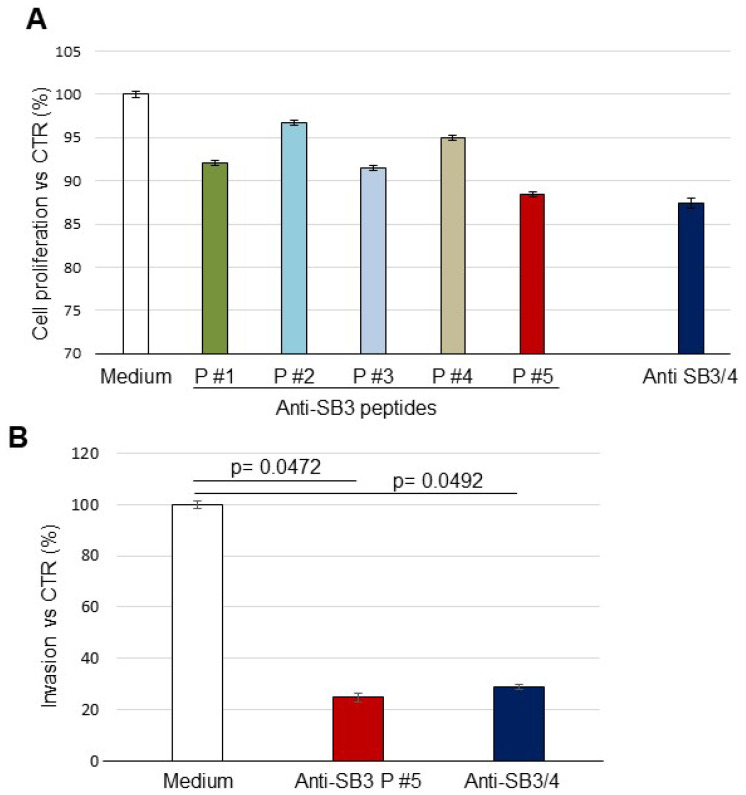
Proliferation and invasion assays. (**A**) Proliferation assay detected by the xCELLigence DP instrument in presence or in absence of the different antibody preparations. The results are expressed as means ± SD of the ratio between the cell index obtained in the HepG2/SB3 cell line and in the control HepG2 cells. No significant differences on proliferation rate were observed. (**B**) Invasion assay results obtained using the system’s CIM plate of the xCELLigence system, where Matrigel was used as a solid matrix. The results are expressed as means ± SD of the ratio between values obtained in HepG2/SB3 cells and in HepG2 control cells.

To investigate whether the anti-SB3 peptide preparations were able to interfere with the main biological activities of the serpin [31], we assessed cell proliferation in HepG2/SB3 cells overexpressing SerpinB3 and in HepG2/CTR cells in the presence or not of each antibody. As shown in Figure 6A, the antibody with the highest inhibitory activity was anti-P#5, which reduced by 12% the gain in cell proliferation compared to control cells, a result comparable to that obtained using the reference anti-SB3/4 antibody. Trivial modifications in cell proliferation were achieved by the remaining anti-peptide antibodies. Analyzing the ability of cell invasiveness induced by SB3, a 75% reduction in the gain of invasiveness was achieved in HepG2/SB3 cells using anti-P#5 antibody, a result similar to that observed with the reference anti-SB3/4 antibody (Figure 6B), while the other antibody preparations did not modify the invasion capability induced by SB3 (data not shown).

## 4. Discussion

The relevance of SerpinB3 in the progression of liver disease is currently well documented by several clinical studies. Reliable evidences show that SB3/4 expression and serum levels of the immunocomplexed isoform (SCCA-IgM) progressively increase during hepatocarcinogenesis, in particular from chronic liver disease to dysplastic nodules to hepatocellular carcinoma (HCC) [27,35,36]. Several studies support the potential clinical value of the SB3/4 detection in serum as diagnostic or prognostic markers; however, some studies show conflicting results, leading to controversy on the clinical use of this biomarker [27]. It should be noted that common methods used to detect SB3 have failed to specifically measure its levels, due to the high homology with the SB4 isoform and the poor specificity of utilized monoclonal antibodies, for cross-reactivity reasons. In addition to being considered biomarkers, SB3 and SB4 have been found to associate with several oncogenic processes and therefore can be labeled as oncoproteins. It is worth noting that SB3 and SB4 show a high degree of homology, but they differ mainly in the catalytic site, leading to inhibition of different proteases [3]. The main oncogenic feature of SB3 includes the induction of cell proliferation and epithelial–mesenchymal transition (EMT), leading to cell transformation, migration and resistance to apoptotic cell death [37]. The protection from apoptotic cell death occurs in different ways: through a direct inhibitory effect on JNK and p38 [38,39], by direct interaction with complex 1 of the respiratory chain and inhibition of mitochondrial pore opening [37] or through its protective activity against lysosomal injury, maintaining the integrity of these organelles [40,41]. EMT-promoting activity of SB3 is of particular clinical relevance, given that SB3 overexpression correlates with high-grade, poorly differentiated forms of cholangiocarcinoma [24] or with vascular invasion and metastasis in colon cancer [26]. Therefore, targeted inhibition of SB3, especially of the catalytic site, may help to control cell proliferation and metastasis formation. This approach is facilitated by the fact that SB3 is secreted into the circulatory system and serum levels correlate with disease progression, at least for liver disease. In our study, within the anti-SB3 peptides we have generated, the anti-P#5 antibody provided the best specific reactivity for human SB3 and was directed against the reactive site loop of the protein. This antibody preparation was able to reduce cell proliferation and to strongly inhibit cell invasiveness. The fact that anti-P#5 antibody recognized the reactive site loop of SB3 only at nuclear level, at variance with the other generated antibodies, deserves further consideration. It is well known that SB3/4 is localized predominantly in the cytosol; however, it has been also detected in other subcellular compartments, including the nucleus, both in cells irradiated with UV rays [38] and in the liver of mice transgenic for SerpinB3 [42,43]. In addition, in clinical samples, nuclear SB3/4 has been reported in various cancers [44,45,46], besides cytosolic localization. Further studies are needed to assess the clinical or prognostic value of specific SB3 detection at the nuclear level.

In the last few years, in cancer immunotherapy, increasing evidence indicates that targeting specific biologically active epitopes is of paramount importance for molecular targeting therapy [47], immunotherapy [48], and immunocheckpoint blockade [49,50,51]. For instance, PD-1/PDL-1 pathway blockade has shown promising efficacy in various malignant tumors [52,53] and as many as five drugs targeting this axis have been approved by the FDA for cancer treatment [54]. As recently demonstrated, fibrinogen-like protein 1/lymphocyte-activation gene 3 (FGL1/LAG-3) may be another promising immunocheckpoint [55,56]. The inhibition of poly(ADP-ribose) glycohydrolase (PARG) upregulated in HCC tissue in combination with anti-PD1 antibody is another novel strategy assessed in preclinical models of HCC [57].

In conclusion, our study demonstrates that the generation of epitope-specific antibodies against SB3 could be a useful tool for targeting biologically active sites of this serpin. In particular, targeting the reactive site loop of SB3 could be a novel strategy to block the pro-oncogenic properties of this serpin. Along these lines, the anti-P#5 antibody could become a relevant tool in basic and clinical research, although additional studies are needed to further support our findings.

## Figures and Tables

**Table 1:** Characteristics and amino acid sequences of the five peptides synthetized after identification of immunogenic human SB3 epitope regions and identity with mouse SB3(Sb3a).

SB3 Peptide (AA Position)	Peptide Amino Acid Sequence	Identity (%/aa Overlap)
#1 (16–30)	Cys-βAla-F_1_QQFRKSKENNIFYS_15_	60.0/15 aa (16–29:1–15)
#2 (140–145)	Cys-βAla-P_1_EESRKKINSWVESQ_15_	92.9/14 aa (138–151:2–15)
#3 (268–279)	Cys-βAla-E_1_WTSLQNMRETR_12_	54.5/11 aa (265–275:1–11)
#4 (191–209)	Cys-βAla-K_1_EDTKEEKFWPNKNTYKS_18_	64.7/17 aa (189–205:1–17)
#5 (340–368)	G_1_AEAAAATAVVGFGSSPTSTNEEFHCNHP_29_	80.0/10 aa (338–347:1–10)

## Data Availability

The data presented in this study are available on request from the corresponding author.

## Supplementary Materials

The following supporting information can be downloaded at: https://www.mdpi.com/article/10.3390/biom13050739/s1, Figure S1: Characteristics of murine Serpinb3a sequence; Figure S2: Dot blot results.
