# Supplementary material for: Epitope-Specific Anti-SerpinB3 Antibodies for SerpinB3 Recognition and Biological Activity Inhibition
Source: Biomolecules. 2023 Apr 25;13(5):739. doi: 10.3390/biom13050739 (PMC10216589; doi:10.3390/biom13050739)
Supplement: Supplementary file 1 [file biomolecules-13-00739-s001.zip › biomolecules-2267258-supplementary.pdf]

biomolecules

MDPI

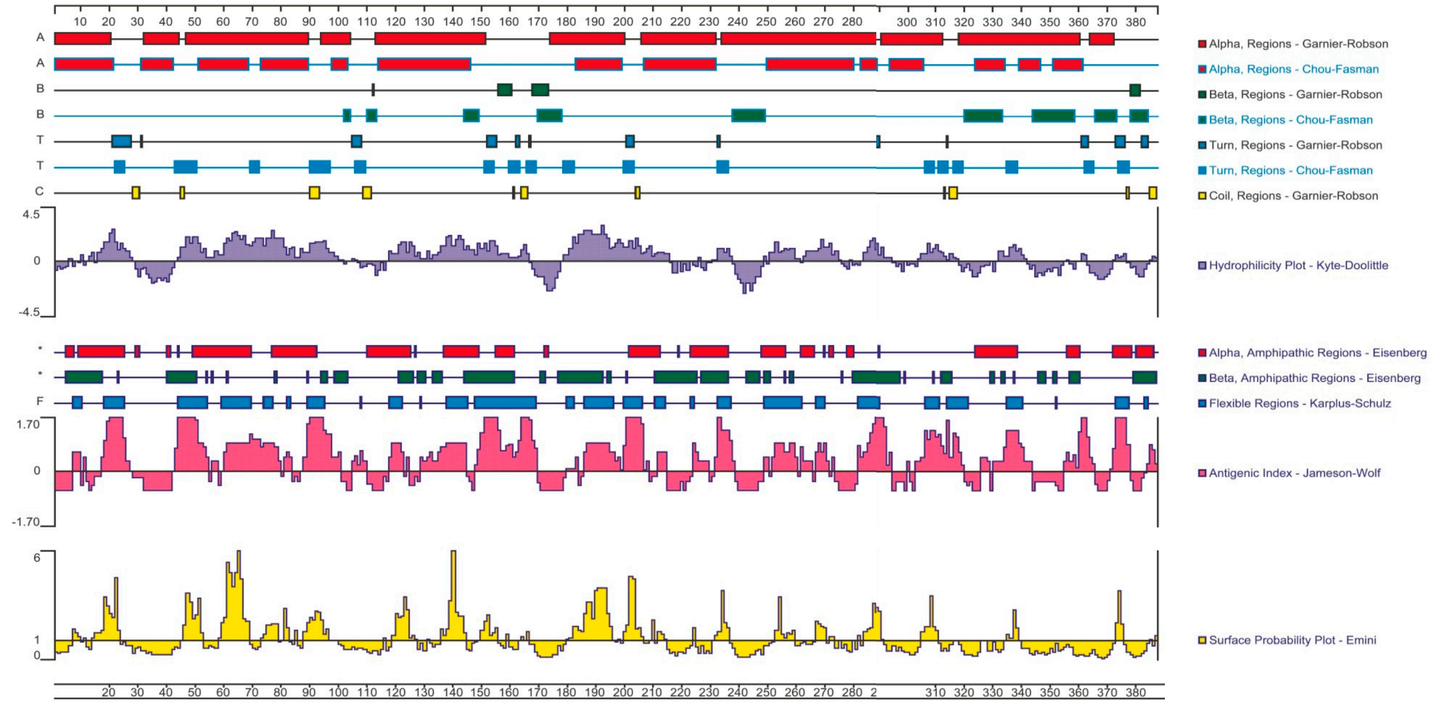

Figure S1. Characteristics of murine Serpinb3a sequence (Uniprot: Q8BG86).

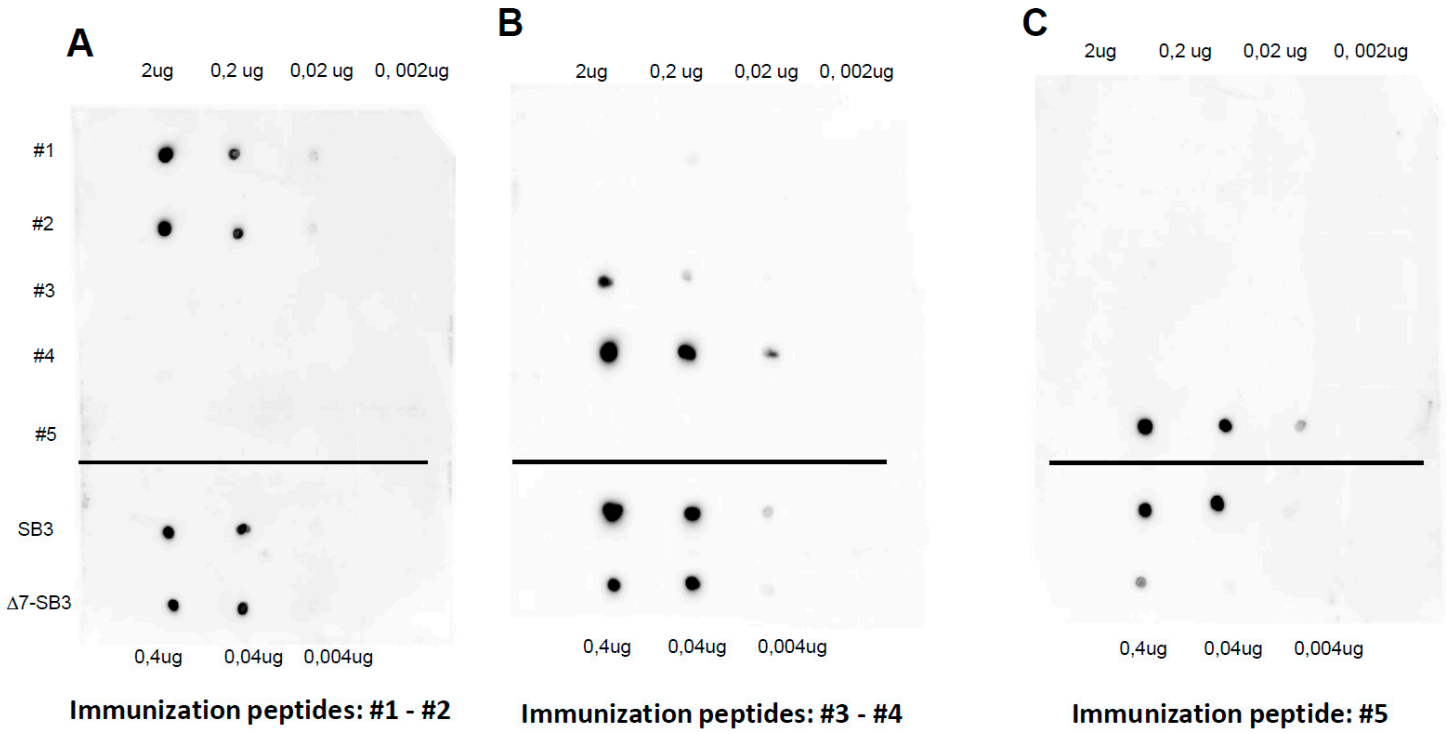

Figure S2. Dot blot results using different concentrations of peptides #1 – #5, recombinant SerpinB3(SB3) and SerpinB3 deleted in the reactive site loop (Δ7-SB3) in the solid phase. The three different blots were incubated with anti-P#1 and anti-P#2 (**A**), with anti-P#3 and anti-P#4 (**B**) or with anti-P#5 (**C**).
